# Cardiologists' Perceptions of Cardiogenetic Testing and Management

**DOI:** 10.1016/j.jacadv.2025.101910

**Published:** 2025-07-04

**Authors:** W.H. Wilson Tang, Quan M. Bui, Allison L. Cirino, Lisa Dellefave-Castillo, Brendan J. Floyd, Alejandra Guerchicoff, Marianna Guerchicoff, Amit V. Khera, Joshua W. Knowles, Kristen Lafayette, Andrew P. Landstrom, Daria W. Ma, Ana Morales, Kate M. Orland, Daniel E. Pineda-Alvarez, Siddharth K. Prakash, Paul Theriot, Melissa Dempsey

**Affiliations:** a Heart Vascular and Thoracic Institute, Cleveland Clinic, Cleveland, Ohio, USA; b Divison of Cardiovascular Medicine, Department of Medicine, University of California San Diego, La Jolla, California, USA; c Institute of Health Professions, Massachusetts General Hospital, Boston, Massachusetts, USA; d Center for Genetic Medicine, Feinberg School of Medicine, Northwestern University, Evanston, Illinois, USA; e Division of Cardiology, Department of Pediatrics, Stanford University School of Medicine, Stanford, California, USA; f Tata Consultancy Services, New York, New York, USA; g Division of Pediatric Arrhythmia and Electrophysiology, Italian Hospital of Buenos Aires, Buenos Aires, Argentina; h Department of Medicine, Harvard Medical School, Boston, Massachusetts, USA; i Verve Therapeutics, Boston, Massachusetts, USA; j Division of Cardiovascular Medicine, Department of Medicine, Brigham & Women's Hospital, Boston, Massachusetts, USA; k Division of Cardiovascular Medicine, Department of Medicine, Cardiovascular Institute and Prevention Research Center, Stanford University School of Medicine, Stanford, California, USA; l CardioGenomic Testing Alliance, Washington, District of Columbia, USA; m Division of Pediatric Cardiology, Department of Pediatrics, Duke University School of Medicine, Durham, North Carolina, USA; n Smidt Heart Institute, Cedar Sinai Medical Center, Los Angeles, California, USA; o Department of Genomic Health, Geisinger, Danville, Pennsylvania, USA; p Division of Cardiovascular Medicine, Department of Medicine, University of Wisconsin-Madison, Madison, Wisconsin, USA; q Labcorp Genetics Inc, San Francisco, California, USA; r Department of Internal Medicine, The University of Texas Health Science Center at Houston, Houston, Texas, USA; s American College of Cardiology, Washington, District of Columbia, USA; t Illumina Inc, San Diego, California, USA

**Keywords:** cardiogenetic testing, cardiovascular genetics, competency, genetic counseling, implementation

## Abstract

**Background:**

Cardiogenetic testing has become clinically relevant as genetic insights increasingly contribute to the understanding and management of cardiovascular diseases of genetic origin. However, utilization of cardiogenetic testing remains variable and underutilized.

**Objectives:**

The purpose of this study was to assess cardiologists' perceptions of cardiogenetic testing and identify relevant barriers, facilitators, educational needs, and clinical applications.

**Methods:**

We surveyed 161 cardiologists using the American College of Cardiology CardioSurve Panel between March and April 2024.

**Results:**

Among respondents, 80% reported that they have directly ordered or facilitated a referral for cardiogenetic testing for their patients. Generally, cardiologists from our testing group felt confident identifying and referring patients for testing, but only 40% confidently ordering tests and only 31% were confident interpreting results. A substantial portion of respondents (40%) had not received any training in cardiogenetic testing. Furthermore, 76% of those who had never ordered testing did not receive relevant education in cardiogenetic testing. The majority (59%) had access to genetic counselors though this was limited for those less familiar with testing. Common barriers included perceived high cardiogenetic testing costs (60%), limited access to genetic counselors (59%), and lack of confidence in interpreting results (43%). Respondents had substantial variability in perceived insurance coverage. Guidelines and resources from professional societies were top educational tools, whereas most cardiologists (91%) expressed interest in further education in patient selection, testing procedures, and results interpretation.

**Conclusions:**

The survey suggest that improved access to genetic counselors and professionals, clearer guidelines, and expanded education could boost cardiogenetic testing adoption and integration into cardiovascular care.

Cardiovascular disease remains the number one cause of death worldwide with many conditions having a strong underlying etiology.^1^^,^^2^ Advances in human genetics are improving the understanding of a variety of inherited cardiovascular diseases. Cardiogenetic testing is growing in clinical applications to diagnose a number of cardiac conditions, including cardiomyopathies, channelopathies, aortopathies, dyslipidemias, and congenital heart defects. Precision diagnosis of conditions with genetic causes may enable more efficient management, appropriate use of emerging targeted therapies, and follow-up of at-risk family members. However, despite technological advancements and scientific statement recommendations from leading cardiology professional societies,^2^, ^3^, ^4^, ^5^, ^6^ utilization of genetic testing remains low among cardiovascular care providers.^7^^,^^8^ Therefore, an improved understanding of practitioner perceptions and barriers is essential for clinicians to integrate guideline-directed genetic testing into clinical practice.

The objectives of the survey were to assess cardiologists': 1) level of confidence in identifying appropriate candidate patients, ordering cardiogenetic testing, and interpreting genetic test findings; 2) attitudes and beliefs toward incorporating genetic testing into routine cardiovascular care; 3) perceived barriers and facilitators in adoption and recommendation of cardiogenetic testing; 4) knowledge level about the logistical and clinical aspects of cardiogenetic testing; and 5) gaps in education related to cardiogenetic testing and preferences for additional education. Herein, we report the findings of this survey.

## Methods

### Study governance

The survey was conducted by the CardioGenomic Testing Alliance (CGTA) in partnership with American College of Cardiology (ACC). The CGTA was launched in 2022 as a not-for-profit collaborative alliance comprised of leading genomics companies and laboratories that aims to raise awareness and utilization of genomic testing in cardiology.

### Study population

In 2024, CGTA partnered with ACC to conduct a survey through CardioSurve, a select panel of ACC members who provide insights and perspectives on the issues that affect today's cardiovascular professionals. The ACC CardioSurve Panel consists of active Fellows and Associate Fellows of the ACC who voluntarily agree to participate in monthly research surveys for a 2-year term. The composition of the CardioSurve Panel was stratified and modeled via demographic information to ensure representation of ACC's database at the time of selection.

### Survey methodology

This survey was developed following 2 rounds of discussions in January 2024 by CGTA stakeholders including genetic testing company representatives and volunteer scientific advisors who are expert clinicians from various tertiary care hospitals in the United States who routinely manage patients with cardiovascular genetic diseases. The survey, based on the literature and experience of the CGTA advisors, consisted of 18 questions designed to gather a deeper understanding from cardiologists of their perceptions of cardiogenetic testing for cardiovascular patients, with a specific focus on genetic testing behaviors and beliefs, along with the barriers and facilitators to ordering cardiogenetic testing (Supplemental Table 1). The survey was conducted by ACC staff, including survey programming, fielding, and analysis, and was independent from the authors, sponsor, or any other party. Before distribution, the survey was reviewed by collaborating ACC staff with experience and expertise in survey design, and the survey was finalized and revised by stakeholders in February and March 2024 prior to launch. Participant-specific links to the survey with up to 3 reminders were emailed to panel members between March 8, 2024, and April 11, 2024, and all panel members were invited to participate via email. Responses were collated and analyzed descriptively by the ACC Market Intelligence office. Data were descriptively presented as proportions with percentages, and Fisher exact tests were used to determine significant associations between categorical variables. Responses were analyzed using the Statistical Package for the Social Sciences (SPSS) version 27 (SPSS). Advarra Institutional Review Board (IRB) Services reviewed the study protocol and using the Department of Health and Human Services regulations found at 45 CFR 46.104(d),^2^ the IRB determined that the research project is exempt from IRB oversight.

## Results

### Baseline characteristics

A total of 161 of 540 panelists (overall response rate of 30%) completed the survey, and all respondents reported that they are currently treating patients in their practice from a broad range of subspecialties, locations, patient populations, and work settings. Table 1 outlines the characteristics of respondents surveyed (comparisons with nonrespondents are provided in Supplemental Table 2). Respondents reported that they cared for patients with coronary heart disease (62%), heart failure (80%), cardiomyopathies (83%), congenital heart diseases (40%), and arrhythmias (63%). Among the 161 respondents, 128 (80%) reported that they have directly ordered or facilitated a referral for cardiogenetic testing for their patients (defined as referral to cardiogenetic specialists, such as genetic counselors, prior to test ordering), with the majority (89%) of those having done this at least once in the past 12 months. Specifically, 49 (30%) referred to someone else to order/supervise the testing, 63 (39%) ordered the test themselves, and 16 (10%) have done both. Forty percent of respondents reported that they had not received any education or training for genetic testing or cardiogenetics. Among those who had never ordered cardiogenetic testing, 76% had not received education/training. For those that received education/training (60%), 26% had hands-on experience, 24% participated in continuing education courses, and 16% participated in genetics sessions or workshop during conferences, while 12% participated in general online courses or web-based training. Only 4% had formal education or fellowship in clinical genetics.

**Table 1:** Characteristics of CardioSurve Survey Respondents

	All Respondents (N = 161)	Ordered/Referred Cardiogenetic Testing (n = 128)	Never Ordered Cardiogenetic Testing (n = 33)
Male, n (%)	123 (76%)	96 (75%)	27 (82%)
Female, n (%)	38 (24%)	32 (25%)	6 (18%)
Race/ethnicity, n (%)			
White	90 (56%)	71 (56%)	19 (58%)
Asian/Pacific Islander	61 (38%)	52 (41%)	9 (27%)
Hispanic/Latino	5 (3%)	4 (3%)	1 (3%)
Black	5 (3%)	3 (2%)	2 (6%)
Other/Declined to answer	6 (4%)	3 (2%)	3 (9%)
Time in practice, y			
Early career (1-7 y)	61 (38%)	52 (41%)	9 (27%)
Mid-career (8-21 y)	42 (26%)	33 (26%)	9 (27%)
Late career (≥22 y)	57 (35%)	42 (33%)	15 (46%)
Not applicable	1 (1%)	1 (1%)	0 (0%)
Board certification, n (%)			
Electrophysiology	15 (9%)	14 (11%)	1 (3%)
General cardiology	91 (57%)	72 (56%)	19 (58%)
Interventional cardiology	27 (17%)	16 (13%)	11 (33%)
Pediatric cardiology	18 (11%)	18 (14%)	0 (0%)
Other	10 (6%)	8 (6%)	2 (6%)
Primary work setting, n (%)			
Cardiovascular practice	54 (34%)	40 (31%)	14 (42%)
HMO/industry	1 (1%)	1 (1%)	0 (0%)
Hospital	34 (21%)	27 (21%)	7 (21%)
Medical school	54 (34%)	46 (36%)	8 (24%)
Multispecialty group	13 (8%)	12 (9%)	1 (3%)
Other	5 (3%)	2 (2%)	3 (9%)
Geographic region, n (%)			
East	49 (30%)	37 (29%)	12 (36%)
North	36 (22%)	31 (24%)	5 (15%)
South	55 (34%)	41 (32%)	14 (42%)
West	21 (13%)	19 (15%)	2 (6%)
Practice location, n (%)			
Rural	14 (9%)	10 (8%)	4 (12%)
Suburban	53 (33%)	40 (31%)	13 (39%)
Urban	90 (56%)	76 (59%)	14 (42%)
Not applicable	4 (3%)	2 (2%)	2 (6%)
Patients/wk, n (%)			
≤20	15 (9%)	7 (6%)	8 (24%)
21-60	81 (50%)	69 (54%)	12 (36%)
61-100	49 (30%)	41 (32%)	8 (24%)
>100	16 (10%)	11 (9%)	5 (15%)
Practice size (# cardiologists), n (%)			
Large (26+)	75 (47%)	62 (48%)	13 (39%)
Medium (11-25)	38 (24%)	30 (23%)	8 (24%)
Medium small (5-10)	26 (16%)	20 (16%)	6 (18%)
Small (1-4)	19 (12%)	13 (10%)	6 (18%)
Not applicable	3 (2%)	3 (2%)	0 (0%)
Decision-making influence at practice, n (%)			
Influencer	69 (43%)	55 (43%)	14 (42%)
Noninfluencer	88 (55%)	69 (54%)	19 (58%)
Not sure/no answer	4 (3%)	4 (3%)	0 (0%)

Values are n (%).

HMO = health maintenance organization.

### Behaviors, attitudes, and beliefs in cardiogenetic testing

**Figure 1:**
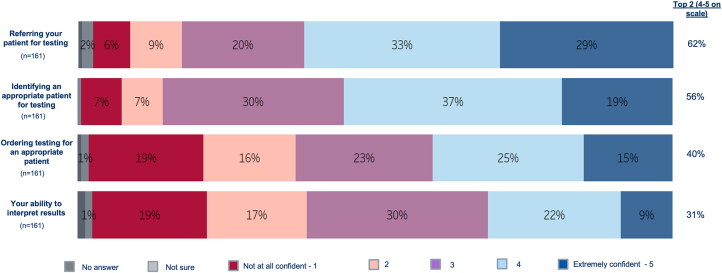
Respondent Confidence With Elements Related to Cardiogenetic Testing Majority of cardiologists surveyed reported that they felt very/extremely confident when referring a patient for cardiogenetic testing (62%) and identifying an appropriate patient for testing (56%). However, less than half reported that they were very/extremely confident when ordering testing for an appropriate patient (40%) and their ability to interpret results (31%).

**Figure 2:**
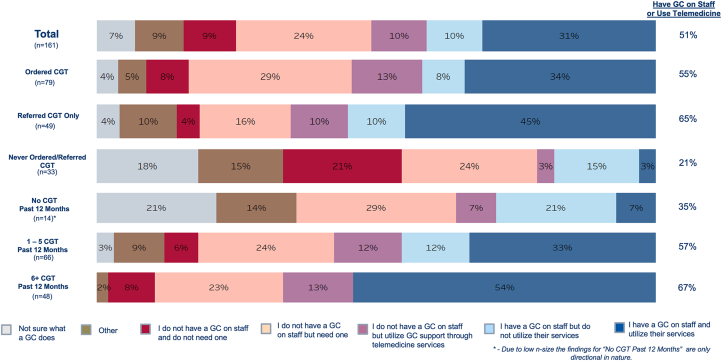
Access to Genetic Professionals and Telegenetics Support When Ordering Cardiogenetic Testing or Interpreting Results Stratified by Testing Experience One-half of cardiologists (51%) reported that they have a GC on staff or utilize GC support services via telemedicine. Across all audiences, at least half of cardiologists reported access to a GC on staff or via telemedicine, except for those who have never ordered/referred CGT (21%) or none in the past 12 months (35%). CGT = cardiogenetic testing; GC = genetic counselor.

Most respondents surveyed reported that they felt very/extremely confident when referring a patient for testing (62%) and identifying an appropriate patient for testing (56%) (Figure 1). However, less than half reported that they were very/extremely confident when ordering testing for an appropriate patient (40%) or being able to interpret the results (31%). Those who had never ordered cardiogenetic testing or who had not ordered within the past year were much less confident than those who had ordered or referred for testing at least once in the last year (*P* < 0.001 for each measure) (Supplemental Figure 1). Over half of the respondents (59%) reported that they had access to genetics professionals that they could consult with when ordering cardiogenetic testing or interpreting results. Access to a genetic counselor on staff or via telemedicine was higher in those who had ordered/referred for at least one cardiogenetic test vs none in the last year (58% vs 35%; *P* < 0.01) (Figure 2). For those who had never ordered/referred cardiogenetic testing, only 21% reported having access to a genetic counselor on staff or via telemedicine (Figure 2).

Respondents believed that the most common cardiac conditions that warranted cardiogenetic testing include hypertrophic cardiomyopathy (89%), long QT syndrome (88%), familial hypercholesterolemia (76%), dilated cardiomyopathy (69%), and thoracic aortic aneurysm (63%). They often referenced recommendations from clinical guidelines or expert consensus statements for management of these conditions. Those who had the most experience with genetic testing (≥6 tests in the past year) tended to be more accurate in their knowledge of the current recommendations for genetic testing of these conditions compared to their colleagues who ordered tests less frequently. Interestingly, those who had not ordered within the past 12 months or had never ordered cardiogenetic testing still believed that patients with the abovementioned conditions should be offered cardiogenetic testing, albeit in lower percentages (Supplemental Figure 2). For example, 82% of those who had never ordered or referred for testing believed that current guidelines recommend testing for hypertrophic cardiomyopathy, a rate not significantly different from those who had ordered testing (*P* = 0.21).

### Knowledge and barriers/facilitators of cardiogenetic testing

**Figure 3:**
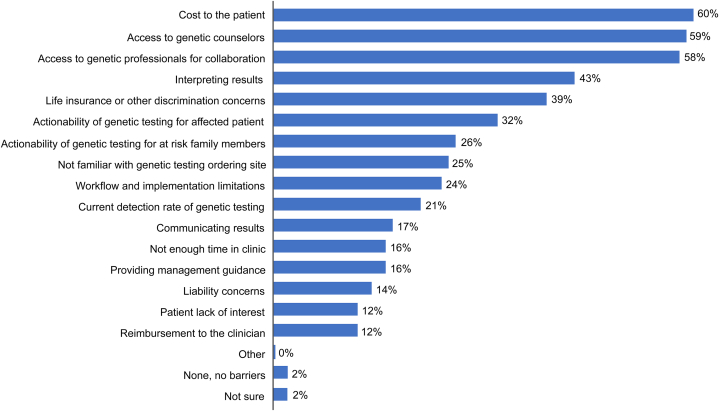
Perceived Barriers to Implementation and Utilization of Cardiogenetic Testing Stratified by Testing Experience Almost all cardiologists (96%) reported at least one barrier to cardiogenetic testing implementation and utilization. The top barriers cited were the cost to the patient (60%), access to genetic counselors or genetics professionals for collaboration (58%-59%), and interpreting results (43%).

**Figure 4:**
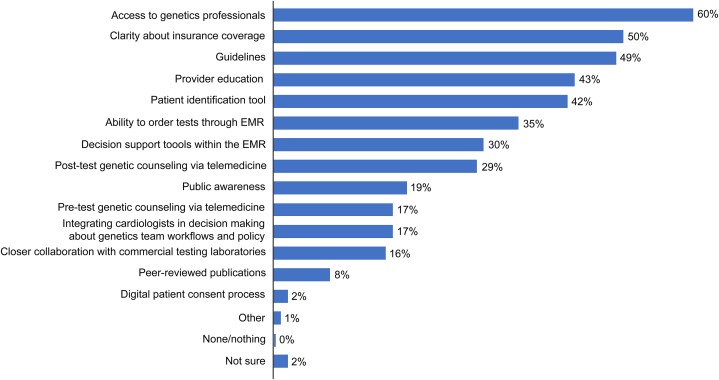
Facilitators to Implementation and Utilization of Cardiogenetic Testing Stratified by Testing Experience Cardiologists indicated that the most helpful elements for supporting the implementation and utilization of cardiogenetic testing were access to genetics professionals (60%), clarity about insurance coverage (50%) and guidelines (49%). EMR = electronic medical record.

**Figure 5:**
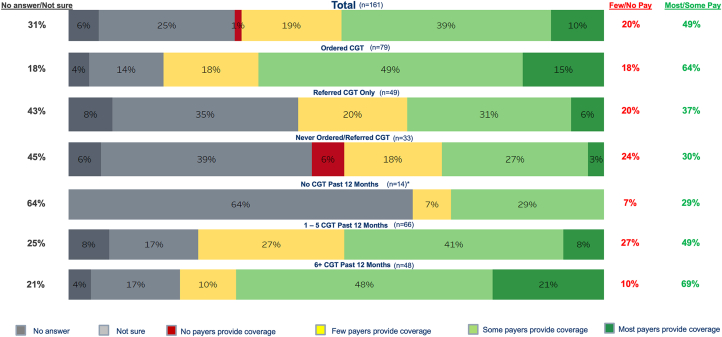
Cardiologists' Perception of Insurance Coverage Landscape for Cardiogenetic Testing Stratified by Testing Experience Cardiologists were divided on their perceptions of the insurance coverage landscape for cardiogenetic testing. Almost one-half (49%) reported that most/some payers provide coverage, one-fifth (20%) indicated that few or no payers provide coverage, and nearly one-third (31%) were not sure or had no answer to the payer coverage aspects of cardiogenetic testing. Abbreviation as in Figure 2.

Almost all respondents (96%) identified at least one barrier to cardiogenetic testing implementation and utilization. Respondents believed that the top barriers to cardiogenetic testing were the perceived high cost to the patient (60%) and access to genetic counselors (59%) or genetic professionals for collaboration (58%) (Figure 3). Most respondents (60%) believe that access to genetic professionals with expertise in cardiovascular genetics would be among the most helpful facilitators to support the utilization of cardiogenetic testing, while tools for patient identification, ordering tests, and decision support within the electronic health record were identified as helpful elements to support implementation and utilization of cardiogenetic testing (Figure 4). Half of the respondents felt that clarity about insurance coverage would be needed to support broad implementation of cardiogenetic testing. Those who had ordered cardiogenetic testing were more likely to know about payer coverage and estimated costs. Overall, respondents estimated average patient cost for a cardiogenetic testing panel being in the range of US $1,355. While respondents were divided in their perceptions of the insurance coverage landscape for cardiogenetic testing, 49% reported that “most/some” payers provide coverage even though 25% were unsure of the cost (Figure 5). Those who had experience with ordering cardiogenetic testing reported that they were more knowledgeable about payer coverage (Figure 5) (*P* < 0.01 compared with those who had referred or never ordered testing).

### Training and education in cardiogenetic testing and management

**Figure 6:**
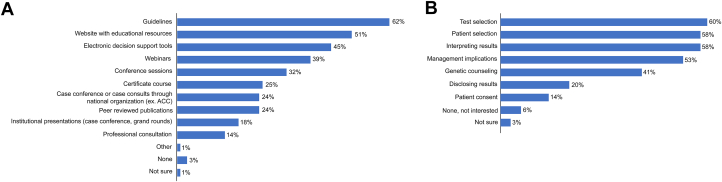
Most Helpful Elements Supporting Implementation and Utilization and Educational Interest in Cardiogenetic Testing (A) Guidelines (62%), website with educational resources (51%), and electronic decision support tools (45%) were considered by cardiologists to be the most useful ACC educational resources on cardiovascular genetics and cardiogenetic testing. (B) Almost all cardiologists surveyed (91%) were interested in at least one area of further education on cardiovascular genetic testing. The topics of greatest interest were test selection (60%), patient selection (58%), interpreting results (58%), and management implications (53%). ACC = American College of Cardiology.

The majority of respondents surveyed (91%) were interested in at least one area of further education on cardiogenetic testing. Guidelines and online educational resources from professional/scientific societies were the top educational resources on cardiogenetic testing and management (Figure 6A). Other helpful educational elements for supporting implementation include webinars and conference sessions. Approximately one-quarter of respondents suggested certificate courses, case conferences, or peer-reviewed publications as helpful educational resources. Topics of greatest interest for further education included test selection, patient selection, and results interpretation (Figure 6B).

## Discussion

Among a contemporary cohort of practicing cardiologists in the United States, we describe the general perceptions of cardiogenetic testing for cardiovascular patients (Central Illustration). While most cardiologists surveyed have directly ordered or facilitated a referral for cardiogenetic testing, they also identified key barriers to testing including unsure of (or perceived high) cost to the patient, access to genetic counselors/professionals with expertise in cardiovascular genetics, and insurance coverage clarity. This survey revealed a care gap between recognition of the clinical importance of cardiogenetics and confidence in adoption of routine cardiogenetic testing. As the respondents were U.S. fellows who are deeply immersed in training, this study reveals a gap in some training programs surrounding cardiogenetics education. However, regardless of training or testing experience, nearly all respondents (91%) indicated a desire to gain competency through further education or training, especially in topics of patient/test selection and cardiogenetic testing results interpretation and management. Specifically, respondents desired education through clinical practice guidelines, training resources, and clinical decision support tools. These findings suggest that cardiologists recognize the importance of cardiogenetic testing to deliver individualized care for patients with cardiovascular genetic diseases but lack the education and tools to do so. This survey highlights the potential to expand cardiogenetic testing through enhancing training with hands-on experiences, increasing access to genetic professionals, and clarifying insurance coverage.

**Central Illustration:**
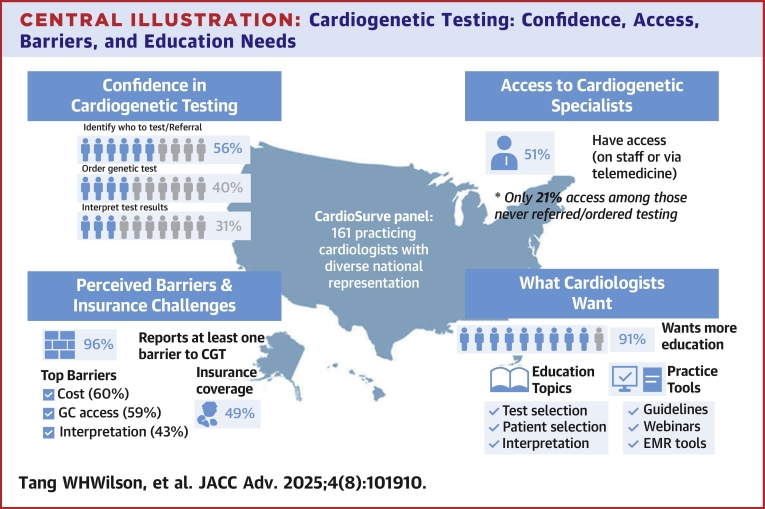
Cardiogenetic Testing: Confidence, Access, Barriers, and Education Needs Abbreviation as in Figures 2 and 4.

An important barrier to ordering cardiac genetic testing is the gap in provider education and confidence.^9^ Prior studies have found that clinicians positively view the use of genetics in the management of cardiovascular diseases, but lack confidence and training.^10^ In a 2021 single-center survey, more than half of nongenetic practitioners reported that they were not confident about the types of cardiogenetic testing available, nor were they confident in the appropriate genetic testing ordering or treatment recommendations based on test results.^11^ In the same study, the most desired topics for an educational program were risk assessment and management of cardiovascular genetic diseases based on guidelines.^11^ These key points were also conveyed in qualitative interviews with cardiovascular providers by the same investigators.^12^ Another study demonstrated that physicians felt somewhat unprepared for widespread whole genome sequencing adoption due to limited genomic knowledge, lack of standards, and time constraints. Specialty-specific concerns highlight the need for targeted education, policy changes, and greater familiarity with results interpretation.^13^ CardioSurve panelists, who uniquely represent a wide spectrum of practicing cardiologists, indicated that they are somewhat confident in identifying appropriate patients but less comfortable with ordering and interpreting cardiogenetic tests, especially without direct experience or support from genetic professionals with expertise in cardiovascular genetics. Respondents were most interested in identifying patients who may have a genetic risk, knowing which genes might be associated with those risks, and managing patients with disease-causing genetic variants. These findings imply that hands-on experience and specific training in cardiogenetics are critical for improving confidence, as respondents with prior education or testing experience reported higher comfort levels across all measures. The lack of training opportunities, highlighted by the fact that 40% of respondents reported not having any education in cardiogenetics, reveals an urgent need for expanded educational opportunities to better equip cardiologists. Preferred formats included professional/scientific society guidelines, online interactive courses, and webinars, suggesting that providing accessible, structured resources on these topics could bridge knowledge gaps and increase confidence in cardiogenetic testing. Many educational resources are available online from professional societies and individual health care institutions although some require fees (Supplemental Table 3).^6^^,^^14^, ^15^, ^16^, ^17^, ^18^, ^19^, ^20^ Perhaps the true barrier is awareness of these existing resources or better tailored options for busy practicing cardiologists that can be interactive or with hands-on experience to be able to generate comfort ordering and interpreting cardiogenetic testing.

A second major barrier is limited access to genetic counselors and other genetic professionals with expertise in cardiovascular genetics. In this survey, only 59% of cardiologists reported access to such professionals with a substantial number relying on telemedicine. Although cardiologists recognize the clinical utility of cardiogenetic testing for cardiovascular diseases of genetic origin, they often rely heavily on genetic professionals for guidance and to execute cardiogenetic testing and interpreting results. For cardiologists who had little or no experience with cardiogenetic testing, access to a genetic counselor was particularly limited, suggesting that enhancing access to genetic professionals could help increase testing among cardiologists with less experience in this area. These access limitations are compounded by cost, policy, and regulatory challenges. Advancing policies that recognize genetic counselors as health care providers to promote sustainable reimbursement and empowering primary care and nursing providers with basic training in cardiogenetic testing and well-defined screening protocols would foster an equitable environment in which at-risk patients can be identified and testing initiated in any clinic, especially where genetic professionals are scarce.^21^ Those who had never ordered genetic testing reported low confidence in their ability to identify patients who would benefit from testing (18% confident), yet this same group correctly noted that current guidelines recommend genetic testing for hypertrophic cardiomyopathy (82%), long QT syndrome (79%), familial hypercholesterolemia (73%), and thoracic aortic aneurysm (55%), each at rates that were not different from respondents who had ordered testing. This may suggest a gap between identifying appropriate patients according to guidelines and their appropriate referral in clinical practice. Robust evidence to support such approaches, validated by clinical data and implementation science, will be key in addressing the gaps highlighted by this survey.

Insurance coverage uncertainty appears to play a significant role in the routine adoption of cardiogenetic testing, even though it may cost less than other routine cardiac procedures or devices. Transparencies in insurance coverage for cardiogenetic testing would facilitate greater access with the potential to be cost-effective as early detection helps prevent costly health complications.^22^, ^23^, ^24^, ^25^ Indeed, public-private partnerships with biotech companies and pharmaceutical firms or research programs have already co-funded genetic testing initiatives, thereby reducing costs for patients and health care systems.^26^^,^^27^ Future efforts should focus on dispelling misconceptions about the cost of genetic testing while addressing equity in access. Emphasizing affordability and integrating equity-driven strategies can ensure broader, fairer access to genetic testing for all populations. For example, integrating cardiogenetic testing into public health programs, like newborn screening in high-risk communities, can provide affordable access, enable early detection of cardiovascular genetic diseases, and reduce long-term health care costs. Community and family-focused education, along with testing discounts, can raise awareness, increase demand, and support informed health decisions. While these perceived cost constraints may not be generalizable to other health care systems that are less dependent on private insurance coverage, barriers identified in implementing cardiogenetic testing remained common challenges.

Cardiogenetic testing is a transformative element of modern cardiology that enhances risk stratification and tailored care and is becoming increasingly relevant in everyday clinical practice as genetic markers are identified that predict disease risk, guide treatment decisions, and shape preventive strategies. Integrating genetic data into cardiovascular care goes beyond routine testing and requires cardiovascular clinicians to enhance their skills in genetic interpretation to support precision medicine. Embracing cardiogenetic testing as a core clinical tool promotes more personalized, patient-centered care. While many health care organizations have dedicated specialty clinics with expertise in managing inherited heart conditions, such approaches may unintentionally limit cardiogenetic services by disincentivizing other clinicians from engaging with cardiogenetics. While specialists remain essential for managing complex cases, broader adoption by all cardiovascular clinicians supports a collaborative model that allows for proactive monitoring and earlier intervention. To build a comprehensive cardiovascular genetics system, future efforts should focus on creating a community of practice that integrates non specialized practice with regional centers of excellence, expanding access to continuing medical education in cardiogenetics, integrating genetics into training programs, securing funding and reimbursement for cardiogenetic services, and advocating for formal recognition of genetic counselors as health care providers by Centers for Medicare and Medicaid Services.

### Study limitations

Using a survey to assess practicing cardiologists' perception of barriers and opportunities in cardiogenetic testing can yield valuable insights, but several limitations should be considered. While the survey was informed by the authors' experience and understanding of the literature on barriers and facilitators to cardiogenetic testing, it did not undergo a formal validation process. As a result, the instrument may have overlooked or not reliably measured relevant individual, organizational, or societal constructs that are important to gain a comprehensive understanding of perceptions about cardiogenetic testing and associated barriers, facilitators, educational needs, and clinical practices. CardioSurve panelists who responded to the survey may already have some interest or experience with cardiogenetic testing, skewing results toward those with more knowledge or positive perceptions of its value. As indicated in the survey findings, respondents' knowledge and experience with genetic testing can vary widely. Those with less familiarity may not fully understand the complexities or may not feel equipped to identify opportunities and solutions accurately. Furthermore, our survey did not delve into why some respondents had lower confidence in interpreting genetic test results, which may be a mix of limited scientific knowledge and/or actual presentation of test results on genetic test reports. This survey also did not directly address the common challenges in cascade genetic testing and clinical screening for at-risk family members once genetic testing has been performed, which continues to be a major gap in our system. It is also important to recognize that this survey captures only clinicians' viewpoints, therefore missing insights into how patients and their families perceive the value, accessibility, and concerns associated with cardiogenetic testing, which are of great value for understanding the real-world effectiveness and challenges. It is also important to recognize that cardiologists provide only one viewpoint of the care team, which may lack insights from other relevant professionals, like geneticists, genetic counselors, primary care physicians, or health care administrators, all of whom have different roles and perspectives on cardiogenetic testing. Cardiologists may be more attuned to clinical or patient-related barriers, potentially overlooking systemic issues such as insurance, data privacy, or organizational support. Nevertheless, this contemporary and diverse survey of practicing cardiologists highlights key opportunities to implement cardiogenetic testing strategies that address access and cost barriers, enabling broader use of its preventive and personalized therapeutic potential (Table 2). As cardiology increasingly intersects with genetics, these measures would not only empower cardiologists to make more informed decisions but also help ensure that patients receive appropriate and timely genetic evaluations for cardiovascular conditions. Addressing these barriers will be essential for expanding the role of cardiogenetics in cardiovascular care and realizing its potential for early detection and personalized treatment.

**Table 2:** Potential Strategies and Tactics to Overcome Barriers to Facilitate Implementation and Utilization of Cardiogenetic Testing

Results	Strategies	Tactics
Cost to the patient	1. Collect information from laboratories about accurate costs for specific indications of and process of providing cost information to patient for cardiogenetic testing 2. Educate physicians on the accurate costs of genetic testing 3. Educate physicians on insurance coverage for genetic testing	1. Create social media content (blog, videos, copy) educating physicians 2. Develop collateral to send to partner and advisor networks outlining the cost/coverage truths 3. Develop campaign with partners that highlight patient stories that can share insurance/cost insight 4. Work with scientific advisors to publish blog on survey data + actual case for cost 5. Encourage payers to have clear and universal medical policies on cardiogenetic testing coverage.
Access to genetic counselors and genetics professionals	1. Educate physicians on GC availability 2. Create tools for better access to GCs 3. Support efforts for CMS coverage of GCs 4. Develop business case for GCs	1. Social media campaign highlighting GCs and how to access 2. Continue to promote and grow “Find a GC Map” in CGTA website 3. Work to develop business case for institutions to see benefit of hiring GC
Interpreting results	1. Educate physicians on how to interpret results 2. Promote current resources on how to interpret results	1. Work with advisors to create educational materials such as online interactive courses 2. Establish regular cadence of Project ECHO meetings to discuss cases 3. Highlight existing materials (Supplemental Table 3)

CGTA = CardioGenomic Testing Alliance; CMS = Centers for Medicare & Medicaid Services; ECHO = Extension for Community Healthcare Outcomes; GC = genetic counselors.

## Conclusions

Despite significant progress over the past decade in educational resources and clinical evidence supporting cardiogenetics, this survey identified a care gap between the recognition of its clinical importance and the confidence in integrating routine cardiogenetic testing. This survey demonstrates heterogeneity in the knowledge and practice of cardiogenetic testing among U.S. cardio logists, identifies support for guideline development, and outlines desired areas for additional education in cardiogenetic test interpretation and management. Overall, these findings imply that cardiogenetic testing could be more widely implemented if supported by improved access to genetic professionals with expertise in cardiovascular genetics, transparency of insurance coverage, and educational initiatives.

Perspectives **COMPETENCY IN MEDICAL KNOWLEDGE:** While most respondents felt comfortable identifying and referring patients for testing, fewer were confident ordering or interpreting tests, with only 40% and 31%, respectively, feeling assured in these areas. Limited training (reported by 40%), high patient costs, and restricted access to genetic counselors (59%) were noted as major barriers.

**TRANSLATIONAL OUTLOOK:** Access to genetics professionals with expertise in cardiovascular genetics and insurance clarity could facilitate broader, more effective adoption of cardiogenetic testing. Continuing education and hands-on training opportunities, designed using implementation science, may further facilitate the uptake of cardiogenetic testing and appropriate management of patients with cardiovascular genetic diseases and their at-risk relatives.

## Funding support and author disclosures

This survey was supported by the CardioGenomic Testing Alliance in collaboration with the American College of Cardiology CardioSurve Panel. Dr Prakash has received funding from 10.13039/100006108National Center for Advancing Translational Sciences and 10.13039/100000051National Human Genome Research Institute to develop a continue medical education course for clinicians about cardiogenetic testing (UL1TR003167-02S2). Dr Tang has served as a consultant for Cardiol Therapeutics, Genomics PLC, Zehna Therapeutics, WhiteSwell, Boston Scientific, CardiaTec Biosciences, Bristol Myers Squibb, Alleviant Medical, Alexion Pharmaceuticals, Salubris Biotherapeutics, BioCardia; and has received honorarium from Springer, Belvoir Media Group, and American Board of Internal Medicine. Dr Bui is a consultant for Papillon Therapeutics and Lexeo Therapeutics. Dr Bui is a current recipient of Janice Wiesman Young Investigator Grant (Ionis Pharmaceuticals) and American Heart Association Career Development Award (24CDA1282533). Dr Khera is an employee of Verve Therapeutics, and has served as scientific advisor to Marea Therapeutics, Allelica, and Google Ventures; and holds equity in Verve Therapeutics, Marea Therapeutics, Color Health, and Allelica. Dr Guerchicoff is an employee of Tata Consultancy Services. Dr Lafayette is an employee of the Conafay Group. Dr Pineda-Alvarez is an employee of LabCorp Inc. Dr Dempsey is an employee and shareholder of Illumina Inc. All other authors have reported that they have no relationships relevant to the contents of this paper to disclose.
