# Supplementary material for: Cardiologists' Perceptions of Cardiogenetic Testing and Management
Source: JACC Adv. 2025 Jul 4;4(8):101910. doi: 10.1016/j.jacadv.2025.101910 (PMC12271069; doi:10.1016/j.jacadv.2025.101910)
Supplement: Supplementary data [file mmc1.docx]

**Supplemental Table 1. 2024 March CardioSurve Survey on Perceptions of Cardiogenetic Testing**

Welcome to the CardioSurve March 2024 Survey!

On behalf of the American College of Cardiology, thank you again for your participation in CardioSurve.

When you complete all of the questions, please be sure to select the "Submit" button.
If you need to exit the survey before you are finished, please select the "Save" button. Your responses will be saved and you can resume taking the survey from the point at which you stopped.

Thank you very much for your time and insights.

To begin...

**1.** Have you ever directly ordered or facilitated a referral for cardiogenetic testing for one of your patients? (answer required)

- Yes, I have ordered
- Yes, I have made a referral
- Yes, I have ordered and made a referral
- No
- Do not treat patients (THANK AND TERMINATE)

This Question is Conditionally Shown if: (1 = Yes, I have ordered OR1 = Yes, I have made a referral OR1 = Yes, I have ordered and made a referral)

**2.** In the past 12 months, for about how many patients have you ordered or referred a cardiogenetic test?

- None
- 1-5
- 6-15
- 16-30
- >30
- Not sure

**3.** Regarding cardiogenetic testing, how confident are you with the following?

|  | Not at all confident 1 | 2 | 3 | 4 | Extremely confident 5 | Not sure |
| --- | --- | --- | --- | --- | --- | --- |
| Identifying an appropriate patient for testing |  |  |  |  |  |  |
| Ordering testing for an appropriate patient |  |  |  |  |  |  |
| Referring your patient for testing |  |  |  |  |  |  |
| Your ability to interpret results |  |  |  |  |  |  |

**4.** Do you have access to a genetics professional you can consult with when ordering cardiogenetic testing or interpreting results?

- Yes, when ordering cardiogenetic testing
- Yes, when interpreting cardiogenetic test results
- Yes, both when ordering and when interpreting cardiogenetic test results
- No
- Not sure

**5.** How are genetic counselors (GC) involved in your institution’s workflow for cardiogenetic testing?

- I have a GC on staff and utilize their services
- I have a GC on staff but do not utilize their services
- I do not have a GC on staff but need one
- I do not have a GC on staff and do not need one
- I do not have a GC on staff but utilize GC support through telemedicine services
- I am not sure what a GC does
- Other, please specify: ____________________

**6.** Which of the following guidelines/expert consensus statements have you referred to in the past year? Please select all that apply.

- 2022 ACC/AHA guideline for the diagnosis and management of aortic disease
- 2022 AHA/ACC/HFSA guideline for the management of heart failure
- 2022 European Heart Rhythm Association (EHRA)/Heart Rhythm Society (HRS)/Asia Pacific Heart Rhythm Society (APHRS)/Latin American Heart Rhythm Society (LAHRS) Expert Consensus Statement on the state of genetic testing for cardiac diseases
- 2020 AHA/ACC guideline for the diagnosis and treatment of patients with hypertrophic cardiomyopathy
- 2018 Clinical genetic testing for familial hypercholesterolemia: JACC scientific expert panel
- 2018 AHA/ACC guideline for the management of adults with congenital heart disease
- 2013 HRS/EHRA/APHRS expert consensus statement on the diagnosis and management of patients with inherited primary arrhythmia syndromes
- 2009 Genetic evaluation of cardiomyopathy—a Heart Failure Society of America practice guideline
- None of the above

**7.** According to current guidelines, cardiogenetic testing should be offered to patients diagnosed with which of the following conditions/ diseases?  Please select all that apply.

- Hypertrophic Cardiomyopathy
- Coronary Artery Disease
- Dilated Cardiomyopathy
- Atherosclerosis
- Stroke
- Long QT Syndrome
- Familial Hypercholesterolemia
- Thoracic Aortic Aneurysm
- Other, please specify: ____________________
- None
- Not sure

**8.** Which of the following do you see as a barrier to the implementation and utilization of cardiogenetic testing?  Please select all that apply.

- Access to genetics professionals for collaboration
- Access to genetic counselors
- Actionability of genetic testing for affected patient
- Actionability of genetic testing for at risk family members
- Communicating results
- Cost to the patient
- Current detection rate of genetic testing
- Interpreting results
- Liability concerns
- Life insurance or other discrimination concerns
- Not enough time in clinic
- Not familiar with genetic testing ordering site
- Patient lack of interest
- Providing management guidance
- Reimbursement to the clinician
- Workflow and implementation limitations
- Other, please specify: ____________________
- None, no barriers
- Not sure

**9.** What do you think the landscape of insurance coverage looks like for cardiogenetic testing?

- Most payers provide coverage
- Some payers provide coverage
- Few payers provide coverage
- No payers provide coverage
- Not sure

**10.** How much, on average, do you think a cardiovascular genetic testing panel costs to the patient today?

- Less than $500
- $500-$1,500
- $1,501-$5,000
- More than $5,000
- Not sure

**11.** Which of the following would you consider to be most helpful to support the implementation and utilization of cardiogenetic testing?  Please select no more than five responses.

- Guidelines
- Public awareness
- Provider education
- Closer collaboration with commercial testing laboratories
- Decision support tools within the EMR
- Ability to order tests through EMR
- Patient identification tool
- Integrating cardiologists in decision making about genetics team workflows and policy
- Digital patient consent process
- Pre test genetic counseling via telemedicine
- Post test genetic counseling via telemedicine
- Access to genetics professionals
- Peer-reviewed publications
- Clarity about insurance coverage
- Other, please specify: ____________________
- None/Nothing
- Not sure

**12.** What level of education or training did you receive for cardiovascular genetics? Please select all that apply.

- Formal education or fellowship in clinical genetics
- Continuing education courses
- Certificate program
- Specific genetics course during higher education
- Genetics sessions or workshops during conferences
- General online courses or web-based training
- Hands on experience
- Other, please specify: ____________________
- I have not received any education or training for cardiovascular genetic testing

**13.** In which of the following areas of cardiovascular genetic testing would you be interested in further education? Please select all that apply.

- Patient selection
- Test selection
- Patient consent
- Genetic Counseling
- Interpreting results
- Disclosing results
- Management implications
- None, not interested
- Not sure

**14.** The ACC is considering the development of educational resources on the topic of cardiovascular genetics and cardiogenetic testing.  Which of the following would be most useful to you on this topic? Please select all that apply.

- Peer reviewed publications
- Guidelines
- Webinars
- Conference sessions
- Website with educational resources
- Certificate course
- Electronic decision support tools
- Professional consultation
- Case conference or case consults through national organization (ex. ACC)
- Institutional presentations (case conference, grand rounds)
- Other, please specify: ____________________
- None
- Not sure

**15.** Finally, please feel free to share any comments you have on any of the topics covered in the survey.

______________________________________________________________

______________________________________________________________

**Supplemental Table 2. Characteristics of CardioSurve Survey Respondents vs Non-Respondents**

|  | **Respondents** | **Non-Respondents** |
| --- | --- | --- |
| **N** | 161 | 379 |
| **Male sex**, n (%) | 123 (76%) | 289 (76%) |
| **Female sex,** n (%) | 38 (24%) | 90 (24%) |
| **Race/ethnicity**, n (%)  White  Asian/Pacific Islander  Hispanic/Latino  Black  Other/Declined to answer | 90 (56%)  61 (38%)  5 (3%)  5 (3%)  6 (4%) | 163 (43%)  149 (39%)  19 (5%)  16 (4%)  36 (9%) |
| **Time in practice**, years  Early career (1-7 y)  Mid-career (8-21 y)  Late career (≥22 y)  Not applicable | 61 (38%)  42 (26%)  57 (35%)  1 (1%) | 131 (35%)  133 (35%)  111 (29%)  4 (1%) |
| **Board certification**, n (%)  Electrophysiology  General cardiology  Interventional cardiology  Pediatric cardiology  Other | 15 (9%)  91 (57%)  27 (17%)  18 (11%)  10 (6%) | 29 (8%)  181 (48%)  120 (32%)  20 (5%)  23 (6%) |
| **Primary work setting,** n (%)  Cardiovascular practice  HMO/industry  Hospital  Medical school  Multi-specialty group  Other | 54 (34%)  1 (1%)  34 (21%)  54 (34%)  13 (8%)  5 (3%) | 170 (45%)  10 (3%)  65 (17%)  86 (23%)  39 (10%)  9 (2%) |
| **Geographic Region**, n (%)  East  North  South  West | 49 (30%)  36 (22%)  55 (34%)  21 (13%) | 37 (29%)  31 (24%)  41 (32%)  19 (15%) |
| **Practice location**, n (%)  Rural  Suburban  Urban  Decline to answer | 14 (9%)  53 (33%)  90 (56%)  4 (3%) | 40 (11%)  127 (34%)  200 (53%)  12 (3%) |
| **Patients/week**, n (%)  ≤20  21-60  61-100  >100 | 15 (9%)  81 (50%)  49 (30%)  16 (10%) | 46 (12%)  144 (38%)  142 (38%)  47 (12%) |
| **Practice size (# cardiologists)**, n (%)  Large (26+)  Medium (11-25)  Medium small (5-10)  Small (1-4)  Decline to answer | 75 (47%)  38 (24%)  26 (16%)  19 (12%)  3 (2%) | 152 (40%)  96 (25%)  67 (18%)  52 (14%)  12 (3%) |
| **Decision-making influence at practice**, n (%)  Influencer  Non-influencer  Not sure/no answer | 69 (43%)  88 (55%)  4 (3%) | 159 (42%)  196 (52%)  24 (6%) |

**Supplemental Table 3. Online Resources for Cardiogenetic Testing**

**The Jackson Laboratory and Northwestern University Feinberg School of Medicine***CME/CNE Course*

[Implementing Cardiogenomics in Clinical Practice](https://education.clinical.jax.org/page/cardiovascular-genetics-education)

- Contains 15 to 30-minute modules that let health care professionals and genetic counselors practice assessing patients’ risk for a genetic cardiac condition. Upcoming modules will address genetic test results interpretation and management of patients with hereditary cardiovascular disease (read the press release [here](https://www.jax.org/news-and-insights/2023/February/the-jackson-laboratory-partners-with-northwestern-to-create-cardiogenomics)).
- Module 1: [Identifying Red Flags and Patterns for Hereditary Cardiovascular Disease](https://education.clinical.jax.org/identifying-red-flags-and-patterns-for-hereditary-cardiovascular-disease-cne)
- Module 2: [Interpreting Positive Results from Cardiac Genetic Testing](https://education.clinical.jax.org/interpreting-positive-results-from-cardiac-genetic-testing-cme)

**The University of Texas Health Science Center at Houston***Certificate Program/CME Course*

[UTHealth Adult Cardiovascular Genomics Certificate Program](https://uthealth.catalog.instructure.com/browse/ms/courses/acgcp)

- Course Overview: The UTHealth Cardiovascular Genomics Certificate program consists of a series of an online educational modules about the genetics and genomics of adult onset cardiovascular diseases. This course is intended for healthcare providers who do not have specialized training in genetics. By focusing on adult cardiovascular genetics, we intend to address disparities in access to genomic education, which have been largely confined to pediatric and cancer genetics programs. The case-based genomics modules may be taken individually for continuing education credit or completed entirely for the stand-alone Cardiovascular Genomics certificate. Furthermore, individuals can also apply these courses toward the MS Degree in Clinical Research.
- Course Objectives: – To fulfill an unmet need for adult cardiovascular genetics education locally, nationally and internationally – To increase awareness of heritable cardiovascular diseases by all health professionals, who are gatekeepers and partners in the diagnosis and treatment of these patients – To improve the recognition and referral of patients with genetically triggered cardiovascular diseases – To innovate genomics education by creating new online platforms and decision support tools for clinicians – To increase the diversity of faculty perspectives by featuring case-based modules that address genomic issues that are relevant to daily clinical cardiovascular practice.

**The American College of Cardiology***Educational Materials*

[Genetic Testing in Cardiology: Increasing Awareness and Understanding](https://learn.acc.org/Page/CARDIOGENOMICS)

- This collection of resources sponsored by the ACC provides materials explaining how to identify inherited cardiac conditions, the utility and best practices of cardiogenomic testing, and the implications of genetic testing on treatment planning for patients and first-degree relatives.
- Materials:
  - [Infographic: Recognizing Mendelian Monogenic Cardiovascular Disease](https://d1oohiyvdehe01.cloudfront.net/acc%2F1%2Ffiles%2F10009%2F13348%2Ffile?Policy=eyJTdGF0ZW1lbnQiOiBbeyJSZXNvdXJjZSI6Imh0dHBzOi8vZDFvb2hpeXZkZWhlMDEuY2xvdWRmcm9udC5uZXQvYWNjJTJGMSUyRmZpbGVzJTJGMTAwMDklMkYxMzM0OCUyRmZpbGUiLCJDb25kaXRpb24iOnsiRGF0ZUxlc3NUaGFuIjp7IkFXUzpFcG9jaFRpbWUiOjE2NzYwNTk0NDV9LCJJcEFkZHJlc3MiOnsiQVdTOlNvdXJjZUlwIjoiMC4wLjAuMC8wIn19fV19&Signature=jLZQL8EphKI9IqpsbiU0bQe3zHxJ-EpO8lplYDnlrkJjAQ0FsqBO9n11v6jbv8dXmq-5sQD1ZPLWmVIm-QrbbwyG3un6hKhltx5zcz7vQMNu9wU0UBiyusLFKHXO572sjmHChfU7E45SrVcLcc-zexPLrpzEoYggOb~FPflfyBU_&Key-Pair-Id=APKAJTOZQY4H2RTLVYOA)
  - [CardiaCast Podcast](https://www.acc.org/latest-in-cardiology#sort=@commonsortdate%20descending&f:@topictermsearchvalues=%5BCardiaCast%5D&f:@articletypecomputed=%5BPodcast%5D): Listen to experts discuss about the benefits of cardiogenomic testing in the context of specific inherited cardiac conditions.
  - [Expert video – Genetics of Cardiovascular Disease](https://acc.bravais.com/s/8N8WpJm8w86i4kQVt8CI)

**CardioNerds***CME Course/Educational Materials*

[CardioNerds – Cardiovascular Genomics Page](https://www.cardionerds.com/cardiovascular-genomics/)

- The CardioNerds Cardiovascular Genomics series is a multi-institutional collaboration made possible by contributions of expert faculty members and research fellows from across the United States. CardioNerds aims to democratize cardiovascular education, promote diversity and inclusion, provide mentorship and sponsorship, and invigorate a love for cardiovascular medicine and science.
- Materials:
  - [CardioNerds Topic Pages](https://www.cardionerds.com/episodes/)
  - [CME curriculum in partnership with VCU Health Continuing Education](https://vcu.cloud-cme.com/course/search?P=4000&search=cardionerds)
  - [Podcast Episodes](https://www.cardionerds.com/episodes/)
  - [“The Heartbeat” Newsletter Signup](https://cardionerds.us4.list-manage.com/subscribe?u=8ad702d2ea6277b6de82d64cb&id=e35539ac55)
  - [Video Case Reports](https://www.cardionerds.com/video-case-list/)
  - [Infographics](https://www.cardionerds.com/infographics/)
  - [Recorded Talks](https://www.cardionerds.com/talks/)

**Beth Israel Deaconess Medical Center for Cardiovascular Genetics Videos**

The Beth Israel Deaconess Medical Center for Cardiovascular Genetics has developed several videos to help patients navigate the world of cardiovascular genetic testing. Click on the below links to find the following informative videos:

- [What is cardiovascular genetic testing?](https://www.youtube.com/watch?v=zY4xJvmayDo&list=PLvvpEAYQHzYZ_LDkSeNL4Zc-2FiS_nch1)
- [What to expect during an appointment](https://www.youtube.com/watch?v=ZGbBuTpgBPA&list=PLvvpEAYQHzYZ_LDkSeNL4Zc-2FiS_nch1&index=2)
- [What to bring to your appointment](https://www.youtube.com/watch?v=2ACbNqewd50&list=PLvvpEAYQHzYZ_LDkSeNL4Zc-2FiS_nch1&index=3)

**American Heart Association’s “From Concept to Practice: A Guide to Cardiovascular Genomics”**

<https://professional.heart.org/en/education/guide-to-cardiovascular-genomics>

The American Heart Association has developed a curriculum program consisting of a series of educational modules on Cardiovascular Genomic and Precision Medicine, a rapidly advancing area of health care. Through these modules, the learner will explore the impact of genomic and precision medicine on cardiovascular health. Continuing education credit will be offered for these courses. Instruction is approximately one hour per module. A certificate of completion will be provided upon successful completion of designated modules and practical sessions.

Modules included:

- Introduction to scope of CV genetics and genomics (Core)
- Counseling, risk assessment in probands and family members (Core)
- Variant interpretation (Core)
- Cardiomyopathies
- Channelopathies and sudden death
- Aortopathies and associated connective tissue diseases
- Familial dyslipidemias
- Pharmacogenomics with an emphasis on clinical utility
- Multifactorially inherited and complex disorders, and polygenic risk scores
- Cardiac disorders with a genetic component and multi-system disorders in which there is cardiac involvement
- Congenital and conditions with presentation in childhood
- Establishing a Specialized Clinical CV Genetics Program (Core)

**Supplemental Figure 1. Respondents’ Confidence with Elements related to Cardiogenetic Testing (CGT) Stratified by Testing Experience**

**
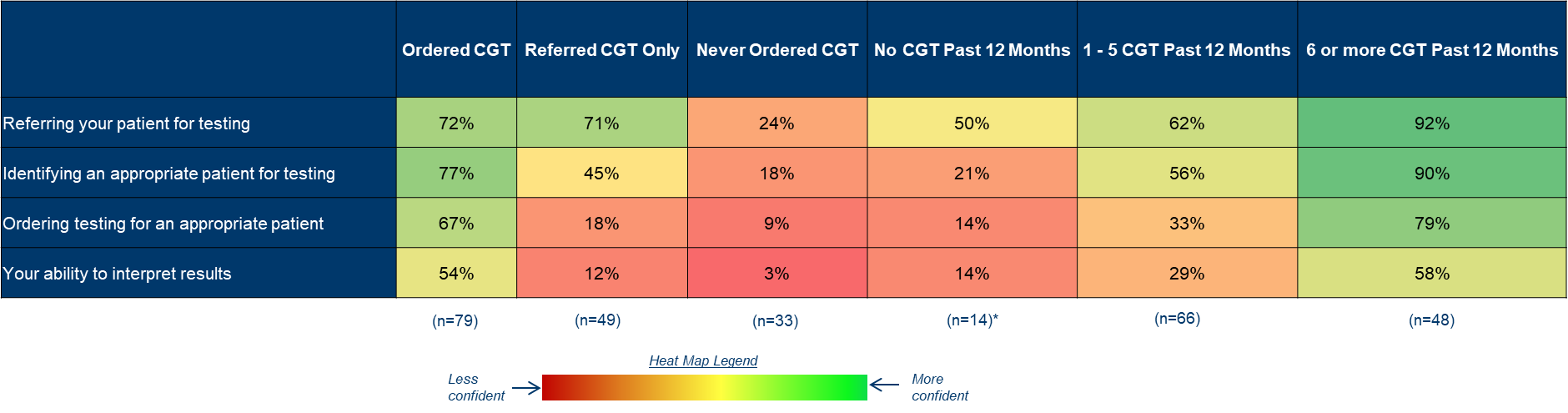
**

Caption: Across the audiences, more than 7 out of 10 cardiologists (71-72%) who have ordered or referred cardiogenetic testing felt very/extremely confident when referring a patient for testing. However, those who have never ordered cardiogenetic testing were less confident in these measures (24%).

**Supplemental Figure 2. Perceived Indications for Cardiogenetic Testing (CGT) for Patients Diagnosed with Specific Conditions/Diseases According to Current Guidelines Stratified by Testing Experience**

**
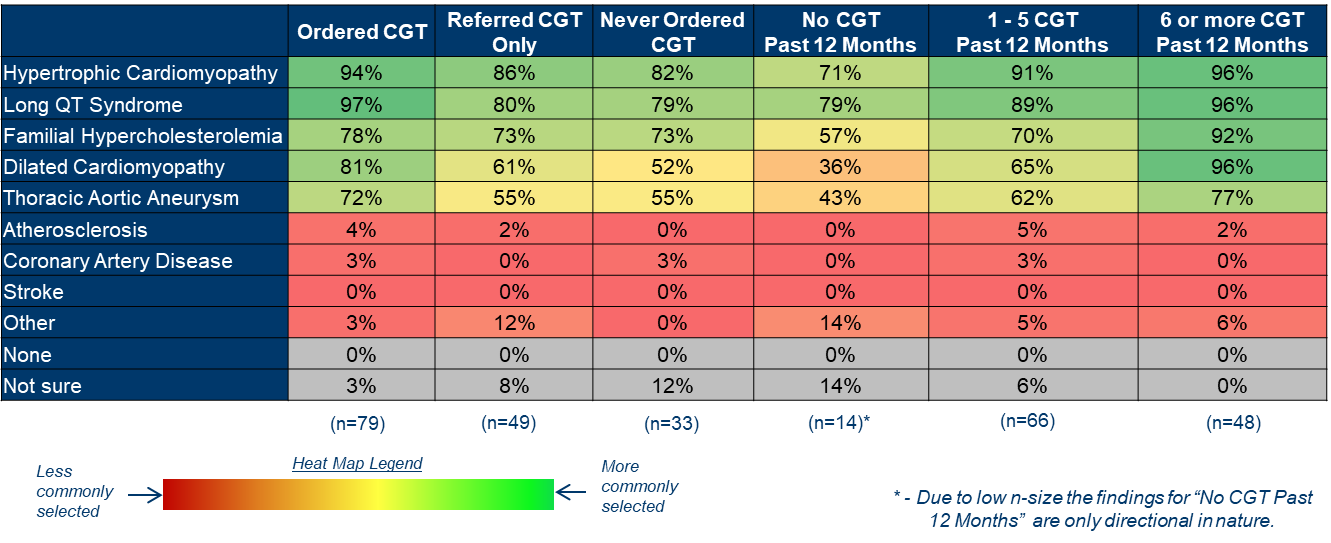
**

Caption: The majority of cardiologists reported that cardiogenetic testing should be offered to patients diagnosed with Hypertrophic Cardiomyopathy (89%), Long QT Syndrome (88%), Familial Hypercholesterolemia (76%), Dilated Cardiomyopathy (69%), or Thoracic Aortic Aneurysm (63%). These were consistently the top responses across the audiences.
